# Examining the secondary impacts of the COVID-19 pandemic on syndemic production and PrEP use among gay, bisexual and other men who have sex with men (GBM) in Vancouver, Canada

**DOI:** 10.1186/s12889-023-17049-w

**Published:** 2023-10-30

**Authors:** Jordan M. Sang, David M. Moore, Lu Wang, Jason Chia, Junine Toy, Julio Montaner, Shayna Skakoon-Sparling, Joseph Cox, Gilles Lambert, Daniel Grace, Trevor A. Hart, Allan Lal, Jody Jollimore, Nathan J. Lachowsky

**Affiliations:** 1 British Columbia Centre for Excellence in HIV/AIDS, Vancouver, Canada; 2 BC Centre on Substance Use, Vancouver, BC Canada; 3 University of British Columbia, Vancouver, Canada; 4 Toronto Metropolitan University (formerly Ryerson), Toronto, Canada; 5 Research Institute of the McGill University Health Center, Montreal, Canada; 6 Direction régionale de santé publique -Montréal, CIUSSS Centre-Sud, Montreal, Canada; 7 Institut national de santé publique du Québec, Quebec City, Canada; 8 University of Toronto, Toronto, Canada; 9 Community Based Research Centre, Vancouver, Canada; 10 University of Victoria, Victoria, Canada

**Keywords:** COVID-19, Syndemic, PrEP, HIV, Gay, bisexual, and other men who have sex with men, Canada

## Abstract

**Background:**

The secondary impacts of the COVID-19 pandemic may disproportionately affect gay, bisexual, and other men who have sex with men (GBM), particularly related to HIV prevention and treatment outcomes. We applied syndemic theory to examine PrEP disruptions during the during the height of the COVID-19 pandemic in Vancouver, Canada.

**Methods:**

Sexually-active GBM, aged 16 + years, were enrolled through respondent-driven sampling (RDS) from February 2017 to August 2019. Participants completed a Computer-Assisted Self-Interview every six months and data were linked to the BC PrEP Program (program responsible for publicly funded PrEP in the province) to directly measure PrEP disruptions. The analysis period for this study was from March 2018-April 2021. We used univariable generalized linear mixed models to examine (1) six-month trends for syndemic conditions: the prevalence of moderate/severe depressive or anxiety symptoms, polysubstance use, harmful alcohol consumption, intimate partner violence, and (2) six-month trends for PrEP interruptions among HIV-negative/unknown GBM. We also applied 3-level mixed-effects logistic regression with RDS clustering to examine whether syndemic factors were associated with PrEP interruptions.

**Results:**

Our study included 766 participants, with 593 participants who had at least one follow-up visit. The proportion of respondents with abnormal depressive symptoms increased over the study period (OR = 1.35; 95%CI = 1.17, 1.56), but we found decreased prevalence for polysubstance use (OR = 0.89; 95%CI = 0.82, 0.97) and binge drinking (OR = 0.74; 95%CI = 0.67, 0.81). We also found an increase in PrEP interruptions (OR = 2.33; 95%CI = 1.85, 2.94). GBM with moderate/severe depressive symptoms had higher odds (aOR = 4.80; 95%CI = 1.43, 16.16) of PrEP interruptions, while GBM with experiences of IPV had lower odds (aOR = 0.38; 95%CI = 0.15, 0.95) of PrEP interruptions. GBM who met clinical eligibility for PrEP had lower odds of experiencing PrEP interruptions (aOR = 0.25; 95%CI = 0.11, 0.60).

**Conclusion:**

There were increasing PrEP interruptions since March 2020. However, those most at risk for HIV were less likely to have interruptions. Additional mental health services and targeted follow-up for PrEP continuation may help to mitigate the impacts of the COVID-19 pandemic on GBM.

**Supplementary Information:**

The online version contains supplementary material available at 10.1186/s12889-023-17049-w.

## Introduction

The COVID-19 pandemic is one of the most significant global public health crises in decades. As of August 6, 2022, there were about 4,100,000 reported COVID-19 cases and more than 43,000 COVID-19-related deaths in Canada; however, these numbers are likely underreported, given increased transmissibility of new COVID-19 variants and lower emphasis on testing and self-isolation for positive cases [1, 2].

Syndemic theory conceptualizes that multiple epidemics interact synergistically, exacerbating one another and contributing to greater disease proliferation [3]. Syndemic theory is a valuable framework for understanding the experiences of gay, bisexual, and other men who have sex with men (GBM) where researchers have found associations between HIV/STI risk [4–6] and established syndemic conditions such as polysubstance use, mental health disorders, intimate partner violence, and alcohol use [7, 8]. Recently, a growing literature has examined associations between syndemic conditions and HIV pre-exposure prophylaxis (PrEP) uptake and adherence among GBM. Tan et al. (2016) argued that screening for syndemic conditions among GBM provides important indicators for PrEP use and adherence [9]. Among young Latino GBM in the United States, Blashill et al. (2019) found that, as syndemic conditions increased, engagement across the PrEP cascade significantly decreased. The authors noted particular concern for psychosocial syndemic conditions such as interpersonal violence (IPV) and polysubstance use, which were negatively associated with PrEP adherence [10]. These findings have been noted in other literature which indicated that greater syndemic conditions have been associated with lower likelihood of PrEP use [11]. However, recent research has also found contradictory results, with odds of PrEP use increasing with a greater number of syndemic conditions among PrEP-eligible Black GBM in the United States [12]. Due to these inconclusive and conflicting findings, further investigation is warranted in this area.

Although most COVID-19 surveillance systems do not collect data on sexual orientation, data from the Behavioral Risk Factor Surveillance System (BRFSS) in the United States found that sexual minority adults have a higher self-reported prevalence of chronic conditions such as cancer, heart disease, kidney disease, HIV and more, which may place these individuals at greater risk of COVID-19 infection as well as increased COVID-19 severity with co-occurring chronic conditions [13]. Apart from the direct impacts of COVID-19, secondary impacts such as social distancing, stay at home orders, and the closure of sexual and mental health clinics may also have negatively impacted the mental and physical health of sexual minority individuals [14]. Starting in mid-March 2020, non-urgent services such as routine sexually transmitted infections (STI) testing or vaccinations were no longer available at sexual health clinic and by August 2020 with gradual lifting of public health restrictions, some began to gradually offer reduced services, while others remained closed [15, 16]. The initial months of the pandemic, saw a decrease in the number of STI tests across the province, including the province’s online option Getcheckedonline [17].

There are concerns regarding how the effects of COVID-19 affected access and adherence to PrEP among HIV-negative GBM and the progress made in preventing HIV transmission [18]. A prospective observational study of GBM in Australia found 41.8% reported discontinuing PrEP due to COVID-19 restrictions. The authors also found PrEP discontinuation was associated with lower odds of receiving an HIV test in the past three months and lower odds of reporting casual sex partners [19]. In France, researchers found 58.8% of GBM reported stopping PrEP use during the first COVID-19 lockdown, with the majority reporting a lack of sexual activity as the main reason for stopping [20]. These findings are aligned with qualitative work from Canada, where GBM reported stockpiling unused PrEP medication and taking PrEP to help alleviate anxiety around HIV, despite reduced sexual activity [21]. A notable limitation of current literature assessing PrEP use and adherence among GBM is the reliance on self-reported data, which is not as accurate as direct-pharmacy data.

Given the newness of COVID-19 and its ongoing effects, further research is warranted to explore the secondary impacts among GBM as they relate to syndemic conditions and PrEP adherence. Building off existing literature, we designed a study to firstly examine trends in the proportion of GBM reporting syndemic conditions and trends of PrEP interruptions using dispensing data from a publicly funded provincial program, from March 2018-April 2021. Next, we assessed correlates between syndemic factors and PrEP interruptions among HIV-negative/unknown GBM in Vancouver. We hypothesized that both syndemic conditions and PrEP interruptions increased after the onset of COVID-19. Further, we hypothesize that a greater number of syndemic conditions will be associated with increased likelihood of PrEP interruptions.

## Methods

Data come from the Vancouver site of the Engage Cohort Study (ECS), a longitudinal biobehavioural cohort study of GBM in Toronto, Montreal, and Vancouver, Canada [22–24]. Vancouver was the only site of the ECS where data linkages were available to a fully funded provincial HIV PrEP program. Baseline data for ECS were collected from February 2017 – August 2019 and participants were recruited using respondent-driven sampling (RDS) with follow-up every six months [25]. The analysis period for this study was from March 2018-April 2021. Of note, PrEP became publicly funded in British Columbia in January 2018. As such, we restricted our data to two years before the COVID-19 pandemic emerged in Canada in March 2020. Further, because of the pandemic, we had no survey data collection from March 2020-September 2020. However, the HIV PrEP program continued to function and collect information throughout the study time period.

Eligibility criteria included: being at least 16 years of age, gender-identifying as a man (including trans men), reporting sex with another man in the past 6 months, currently living in Vancouver, and being able to complete the questionnaire in English. Participants also had to either be a “seed” participant or invited into the study by a previous participant, as per our RDS protocol. Participants completed a questionnaire using computer-assisted self-interview (CASI) which asked questions about sexual behaviours and risk, substance use, psychosocial health, and demographics. For each visit, participants received an honorarium of Canadian dollars (CAD) 50 and an additional compensation of CAD 15 for each eligible recruit who completed a study visit. Full details about the Engage study have been previously reported [22]. All participants provided informed consent to participate and additional consent to have their data linked to the BC PrEP Program. The Engage study was approved by research ethics boards at Toronto Metropolitan University, University of Toronto, St. Michael’s Hospital, University of Windsor, University of British Columbia, Providence Health Care, University of Victoria, Simon Fraser University, and McGill University Health Centre [22].

### Measures

#### PrEP interruptions

PrEP interruptions were measured through data linkages for Engage study participants in Vancouver and the BC PrEP program, which is responsible for the distribution of publicly funded PrEP in the province [26]. Interruptions in PrEP were defined by the BC PrEP Program with the following criteria. First, participants may have formally indicated wanting to stop PrEP to their provider, which then initiated the return of PrEP refill forms to the program. Second, participants who did not refill PrEP for at least 6 months from the date that the last dispensed PrEP would have “run out” (based on once daily dosing) and these individuals were systematically marked as having a PrEP interruption. Analyses focused on PrEP interruptions included participants who had ever used PrEP prior to their interview date, as noted by the BC PrEP Program [27].

#### Syndemic conditions

We included six syndemic conditions in this study. Symptoms of anxiety and depression were measured by the Hospital Anxiety and Depression Scale (HADS) and were dichotomized with scores ≥ 11 equalling moderate/severe and scores 10 or less equalling normal/mild [28]. Polysubstance use was measured through participants self-reporting two or more illicit substances used in the past six months (e.g., ecstasy, crystal meth, crack cocaine, ketamine). Interpersonal violence was measured by the Conflict Tactics Scale, which asks participants about any experiences of IPV. Measurements at baseline include any lifetime experiences of IPV (both perpetration and victimization) and, at follow-up visits, in the past six months [29]. Risk for problematic drinking was measured using the AUDIT-C scale, with scores ≥ 4 indicating high risk for harmful drinking [30]. We also included the sexual abuse questions from the childhood trauma questionnaire to assess childhood sexual abuse (e.g., experiences of being threatened, sexual abuse, sexual touching, coercion into doing sexual things) with responses dichotomized as ever/never [31].

#### Other variables

We included a variable assessing if participants met provincial eligibility for PrEP at their study visit, defined as reporting condomless anal sex in the past six months and any of the following: (1) infectious syphilis or rectal bacterial STI particularly if diagnosed in the past 12 months; (2) use of post-exposure prophylaxis (PEP) more than once; (3) an ongoing sexual relationship with a partner living with HIV with who is not confirmed to be taking HIV treatment and/or has an unsuppressed HIV viral load; or (4) HIV Incidence Risk Index (HIRI) score greater or equal to 10 [26]. Additionally, we asked HIV-negative/unknown participants who indicated PrEP use in the past six months about their PrEP regimen (responses: daily; on-demand; both). We also asked about the number of male sex partners in the past six months and included the Treatment Optimism-Skepticism scale, which measures attitudes towards HIV treatment [32]. Sociodemographic variables include age, sexual orientation, education, current employment, race/ethnicity, annual income, and relationship status. Lastly, we included time as a variable with time dichotomized to before COVID-19 (March 2018-March 2020) and after COVID-19 (September 2020-April 2021).

### Data analysis

**Fig. 1:**
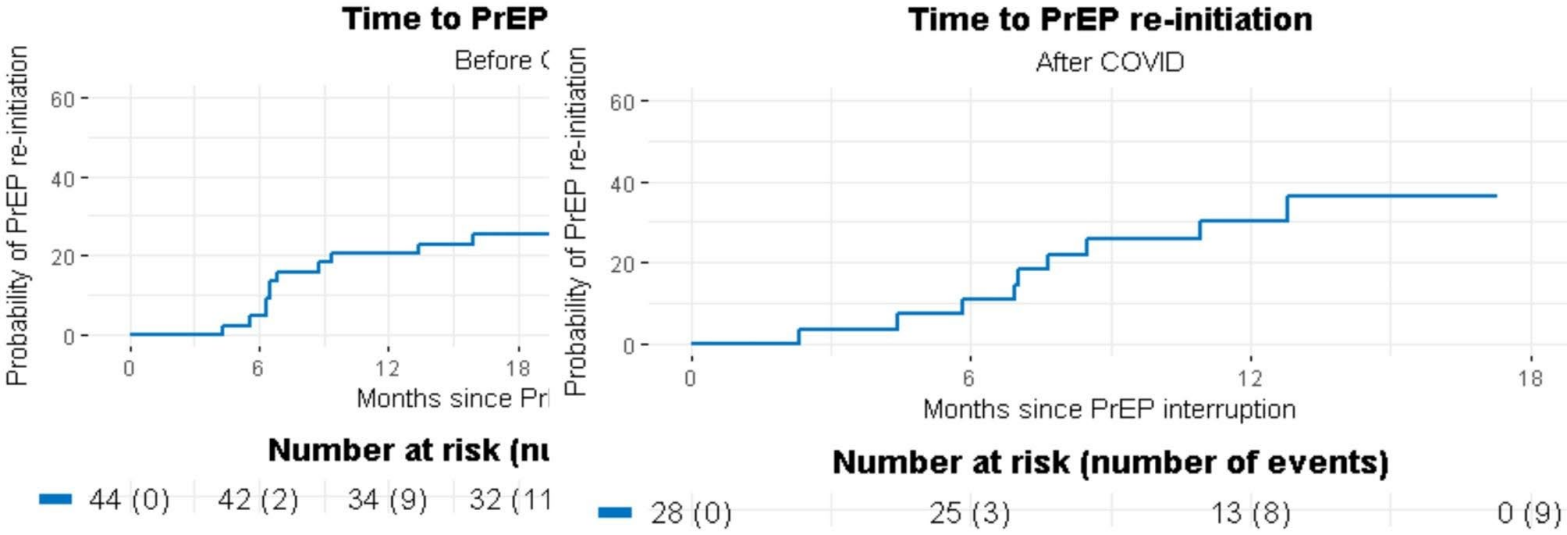
Time to PrEP Restart Before and After COVID among Participants who had ever Experienced a PrEP Interruption (N = 72)

We present demographic data on all participants enrolled in ECS and applied univariable generalized linear mixed models to examine 1) trends in syndemic conditions among all participants. We also include trends in PrEP interruptions using generalized linear mixed modelling to model PrEP interruption with time in six month periods as the independent variable among only those who were HIV-negative/unknown GBM and had ever used PrEP prior to their interview date. Childhood sexual abuse was not included in the syndemic trend analyses as this was a one-time measure and IPV was not included in the syndemic trend analyses since most items asked about lifetime experiences. We applied 3-level mixed-effects logistic regression with RDS clustering (RDS > participants > visit) to examine the individual additive and interaction effects of syndemics on PrEP use among HIV-negative/unknown GBM reporting PrEP use before their study visit. Explanatory variables in the final model were selected based on the Type III *p*-values and minimization of the Akaike information criterion (AIC). The final multivariable model reports adjusted odds ratios (aOR), significance was assessed based on 95% confidence intervals and *p*-value less than 0.05. We also included two Kaplan Meier plots indicating the time from interruption start to end pre-and post-onset of COVID (Fig. 1). Analyses were performed using SAS version 9.4 (SAS, Cary, North Carolina, USA).

We conducted a post-hoc analysis to assess self-reported current PrEP use at their interview date among HIV-negative/unknown GBM reporting PrEP use before their study visit. We applied 3-level mixed-effects logistic regression with RDS clustering (RDS > participants > visit) and explanatory variables in the final model were selected based on the Type III *p*-values and minimization of the AIC.

## Results

A total of 766 participants completed a baseline survey. The median age of participants was 34 years old. Most participants identified as Canadian ethnicity (48.7%), reported an annual income less than $30,000 (46.7%), identified as gay (85.5%), and reported a greater than high school education (84.5%). The majority of participants were also single (55.6%) and were currently employed (73.4%). Full descriptive results can be found in Table 1. Out of the 766 participants, there were 49 who indicated they had moved, 2 who became deceased, 114 who could not be reached for follow up, and 29 who indicated they had dropped out for other reasons. We tested to see if having two or more syndemic conditions was associated with having at least one visit after enrolment. We did not find significant differences between those who were lost to follow up and those who continued with the study.

**Table 1:** Sociodemographic and Syndemic results from Baseline Survey (N = 766)

Sociodemographics	Total N	Median	(Q1-Q3)
Age	766	34	(28–48)
	Total N	N	(%)
**Annual income (CAD)**	766		
Less than 30,000		358	(46.7)
30,000 to 59,999		220	(28.7)
60,000 or higher		188	(24.5)
**Ethnicity**	766		
Canadian		373	(48.7)
Aboriginal		24	(3.1)
European		144	(18.8)
Asian		125	(16.3)
African/Caribbean/Black		9	(1.2)
Mixed Race		17	(2.2)
Another ethnicity		74	(9.7)
**Sexual identity**	766		
Gay		655	(85.5)
Bisexual		37	(4.8)
Another sexual identity		74	(9.7)
**Gender identity**	766		
Cisgender		722	(94.3)
Another gender identity		44	(5.7)
**Highest level of education**	766		
High school or less		119	(15.5)
Greater than high school		647	(84.5)
**Current employment**	766		
No		204	(26.6)
Yes		562	(73.4)
**Current relationship with a main partner**	766		
No		426	(55.6)
Yes		340	(44.4)
**Self-reported HIV status**	766		
Negative/Unknown		575	(75.1)
Positive		191	(24.9)
**Syndemic conditions**			
**HADS Anxiety**	738		
Normal/Mild (score 10 or less)		529	(71.7)
Moderate/Severe (score 11 to 21)		209	(28.3)
**HADS Depression**	736		
Normal/Mild (score 10 or less)		686	(93.2)
Moderate/Severe (score 11 to 21)		50	(6.8)
**Polysubstance use in P6M**	739		
No		417	(56.4)
Yes		322	(43.6)
**High risk of harmful drinking measured by AUDIT-C**	750		
No (Score less than 4)		358	(47.7)
Yes (Score 4 or more)		392	(52.3)
**IPV experiences**	754		
No		353	(46.8)
Yes		401	(53.2)
Other		74	(9.7)
**Experiences growing up as a child: Sexual abuse**	713		
Never		525	(73.6)
Ever		188	(26.4)
**Experiences growing up as a child: Touched**	718		
Never		452	(63.0)
Ever		266	(37.0)
**Experiences growing up as a child: Threatened**	718		
Never		628	(87.5)
Ever		90	(12.5)
**Experiences growing up as a child: Sexual things**	719		
Never		509	(70.8)
Ever		210	(29.2)

Acronyms: HADS = Hospital Anxiety and Depression Scale; P6M = Past Six Months; AUDIT-C = Alcohol Use Disorders Identification Test; IPV = Interpersonal violence; OR = Odds Ratio; CAD = Canadian Dollars

There were 2396 visits from 766 participants between March 2018 to April 2021. Among these, 593 participants had at least one follow-up visit, with a median of 3 follow-up visits over a median of 1.71 years of follow-up. Among syndemic conditions, we did not find a significant trend across the study period in the proportion of participants with moderate/severe anxiety scores (OR = 1.03; 95%CI = 0.94, 1.12). However, we found a trend of increased proportions of participants with moderate/severe depression scores (OR = 1.35; 95%CI = 1.17, 1.56). We also found significant decreases over time in the proportion of participants with polysubstance use (OR = 0.89; 95%CI = 0.82, 0.97), and hazardous drinking (OR = 0.74; 95%CI = 0.67, 0.81). Full results can be found in Table 2.

**Table 2:** Syndemic scores over time from March 2018-April 2021 and Trend Analyses (N = 766 reporting on 2396 study visits)

HADS Anxiety		Calendar Time by every 6 months								
	Mar 2018-Aug 2018			Sep 2018-Feb 2019	Mar 2019-Aug 2019	Sep 2019-Mar 2020	Apr 2020 – Aug 2020	Sep 2020-Feb 2021	Mar 2021-Apr 2021	Total
**Normal/Mild**	263			301	384	405	0	220	149	1722
**Moderate/Severe**	101			82	141	118	0	89	50	581
**Total**	364			383	525	523	n/a	309	199	2303
**%**	27.7%			21.4%	26.9%	22.6%	n/a	28.8%	25.1%	
**OR**	**95% CI**				**p-value**					
1.03	0.94			1.12	0.572					
**HADS Depression**	**Mar 2018-Aug 2018**			**Sep 2018-Feb 2019**	**Mar 2019-Aug 2019**	**Sep 2019-Mar 2020**	**Apr 2020 – Aug 2020**	**Sep 2020-Feb 2021**	**Mar 2021-Apr 2021**	**Total**
**Normal/Mild**	342			357	499	486	0	269	172	2125
**Moderate/Severe**	23			27	27	37	0	42	27	183
**Total**	365			384	526	523	n/a	311	199	2308
**%**	6.3%			7.0%	5.1%	7.1%	n/a	13.5%	13.6%	
**OR**	**95% CI**				**p-value**					
1.35	1.17		1.56		**< 0.001**					
**Polysubstance use in P6M**	**Mar 2018-Aug 2018**		**Sep 2018-Feb 2019**		**Mar 2019-Aug 2019**	**Sep 2019-Mar 2020**	**Apr 2020 – Aug 2020**	**Sep 2020-Feb 2021**	**Mar 2021-Apr 2021**	**Total**
**No**	208		215		304	301	0	190	132	1350
**Yes**	159		176		229	225	0	123	69	981
**Total**	367		391		533	526	n/a	313	201	2331
**%**	43.3%		45.0%		43.0%	42.8%	n/a	39.3%	34.3%	
**OR**	**95% CI**				**p-value**					
0.89	0.82		0.97		**0.007**					
**High risk or Harmful Drinking measured by AUDIT-C**	**Mar 2018-Aug 2018**		**Sep 2018-Feb 2019**		**Mar 2019-Aug 2019**	**Sep 2019-Mar 2020**	**Apr 2020 – Aug 2020**	**Sep 2020-Feb 2021**	**Mar 2021-Apr 2021**	**Total**
**Score less than 4**	166		185		276	304	0	189	132	1252
**Score 4 or more**	201		201		260	232	0	129	72	1095
**Total**	367		386		536	536	n/a	318	204	2347
**%**	54.8%		52.1%		48.5%	43.3%	n/a	40.6%	35.3%	
**OR**	**95% CI**				**p-value**					
0.74	0.67		0.81		**< 0.001**					

Acronyms: HADS = Hospital Anxiety and Depression Scale; P6M = Past Six Months; AUDIT-C = Alcohol Use Disorders Identification Test; IPV = Interpersonal violence; OR = Odds Ratio

Note: Childhood sexual abuse and IPV questions are not included because they were not asked over time

**Fig. 2:**
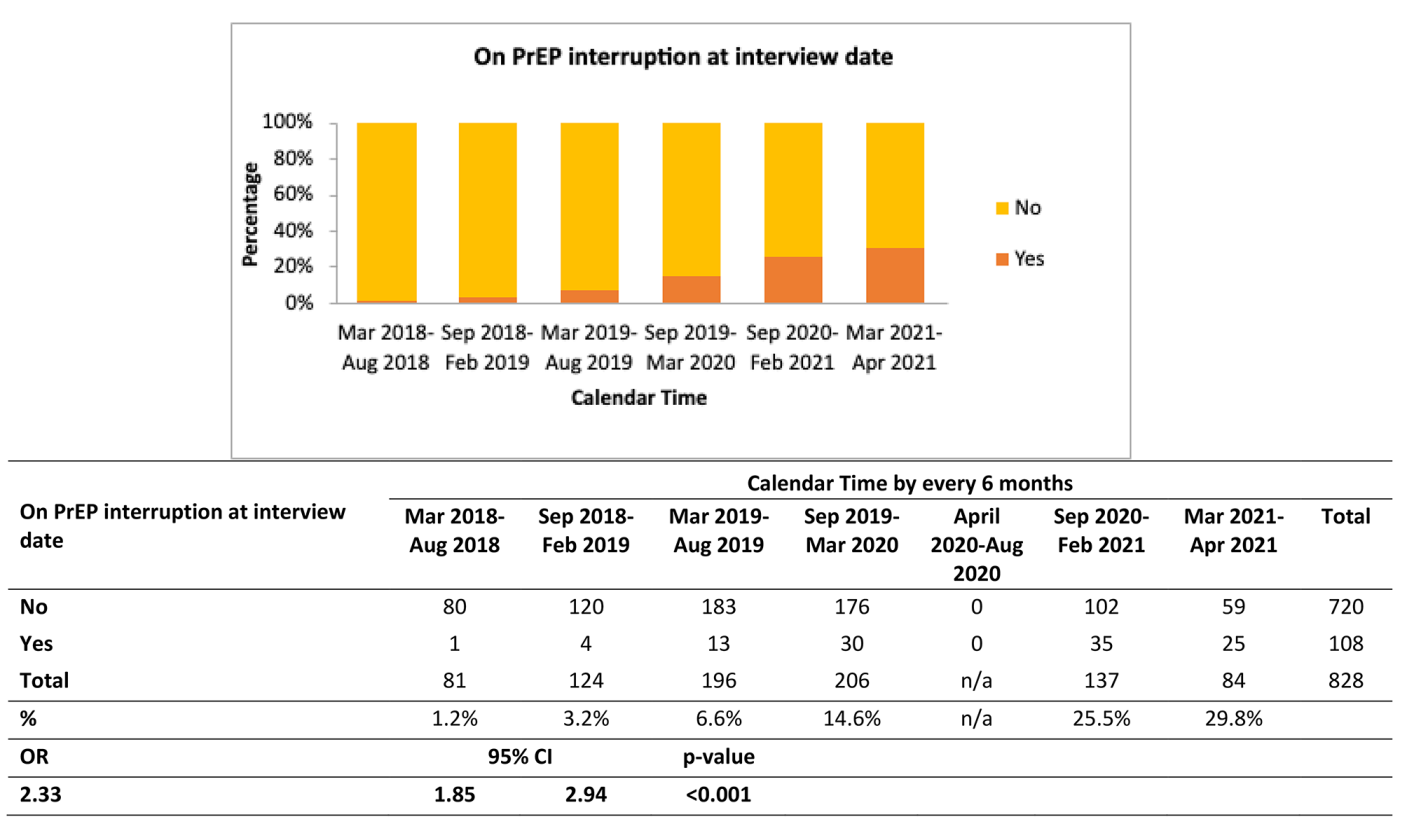
PrEP interruptions from March 2018-April 2021 among HIV negative/unknown GBM who ever Reported PrEP use Prior to Interview Date (N = 280) Footnote: The time between March 2020-September 2020 our offices were closed due to COVID-19 restrictions, thus we had no interviews during this time

Overall, there were 108 visits at which the participants were on a PrEP interruption. Of these, 21 formally indicated they had stopped, and 87 had no refill for at least 6 months. Please note that participants could be on an interruption for more than one visits due to the same PrEP interruption event (e.g., interruption occurred from one visit till the end of follow up). For distinct events (N = 72), there were 15 formal stops and 57 medication lapses. We found a significant increase in the proportion of participants with PrEP interruptions over time, with 1.2% experiencing a PrEP interruption at the start of the study period to 29.8% experiencing a PrEP interruption at the end of the study period (OR = 2.33; 95%CI = 1.85, 2.94), shown in Fig. 2.

Data from 280 HIV-negative/unknown participants who had ever used PrEP before their interview date were used to model the effects of syndemics on PrEP interruptions. The median time between an estimated interruption start date and the first visit during the interruption period was 3.5 months. Self-reported PrEP regimen for first visits (before COVID-19) was 88.8% for daily usage (n = 95), 6.5% reported on-demand use (n = 7), and 4.7% reported both uses (n = 5). Self-reported PrEP regimen for last visits (after COVID-19) was 80.4% for daily usage (n = 86), 12.1% for on-demand (n = 13), and 7.5% for both (n = 8). GBM who had moderate/severe depression scores had greater odds of PrEP interruptions compared to GBM who had normal/mild scores (aOR = 4.80; 95%CI = 1.43, 16.16). GBM who indicated any experiences of IPV had lower odds of PrEP interruptions compared to GBM with experiences of IPV (aOR = 0.38; 95%CI = 0.15, 0.95). Other syndemic conditions were either not significant at the univariable level and/or not selected in the final model (e.g., binge drinking, anxiety, and polysubstance use) (Table 3). We tested interactions between any two syndemic variables, but no interaction terms were significantly associated with the outcome; thus, interactions were not included in the final model.

**Table 3:** Univariable and Multivariable Generalized linear mixed Models Assessing PrEP Interruptions* (N = 280 participants reporting on 828 study visits)

On PrEP interruption at interview date														
		No (N = 720)		Yes (N = 108)		Yes vs. No				Yes vs. No				
Variable	Total N	N	(%)	N	(%)	OR	95% CI		p-value		aOR	95% CI		p-value
**Annual income**	828													
Less than 30,000		199	(27.6)	35	(32.4)									
30,000 to 59,999		264	(36.7)	34	(31.5)	0.70	0.31	1.57	0.383					
60,000 or higher		257	(35.7)	39	(36.1)	0.92	0.38	2.19	0.841					
**Ethnicity**	828													
Canadian		318	(44.2)	49	(45.4)									
Aboriginal		7	(1.0)	1	(0.9)	0.95	0.02	56.35	0.978					
European		173	(24.0)	24	(22.2)	0.69	0.26	1.86	0.461					
Asian		146	(20.3)	17	(15.7)	0.60	0.21	1.75	0.352					
African/Caribbean/Black		8	(1.1)			NA								
Mixed Race		16	(2.2)	5	(4.6)	2.28	0.20	26.48	0.509					
Another ethnicity		52	(7.2)	12	(11.1)	2.06	0.53	8.04	0.298					
**Sexual identity**	828													
Gay		608	(84.4)	91	(84.3)									
Bisexual		26	(3.6)	2	(1.9)	0.64	0.08	5.09	0.676					
Another sexual identity		86	(11.9)	15	(13.9)	0.83	0.31	2.25	0.718					
**Gender identity**	828													
Cisgender		675	(93.8)	101	(93.5)									
Another gender identity		45	(6.3)	7	(6.5)	0.98	0.25	3.80	0.975					
**Highest level of education**	828													
High school or less		49	(6.8)	7	(6.5)									
Greater than high school		671	(93.2)	101	(93.5)	0.77	0.19	3.09	0.707					
**Current employment**	828													
No		130	(18.1)	21	(19.4)									
Yes		590	(81.9)	87	(80.6)	0.82	0.37	1.84	0.629					
**Current relationship with a main partner**	828													
No		408	(56.7)	49	(45.4)									
Yes		312	(43.3)	59	(54.6)	2.13	1.12	4.05	**0.021**		3.58	1.62	7.92	**0.002**
**Time period**	828													
Before COVID (MAR 2018 to MAR 16 2020)		559	(77.6)	48	(44.4)									
After COVID (SEP 2020 to APR 2021)		161	(22.4)	60	(55.6)	9.76	4.81	19.81	**< 0.001**		9.25	4.06	21.1	**< 0.001**
**Overall PrEP eligibility**	822													
No		129	(18.0)	41	(38.3)									
Yes		586	(82.0)	66	(61.7)	0.20	0.10	0.43	**< 0.001**		0.25	0.11	0.60	**0.002**
**Syndemic Conditions**														
**HADS Anxiety**	813													
Normal/Mild (score 10 or less)		533	(75.5)	78	(72.9)									
Moderate/Severe (score 11 to 21)		173	(24.5)	29	(27.1)	1.21	0.60	2.46	0.597					
**HADS Depression**	810													
Normal/Mild (score 10 or less)		653	(92.9)	90	(84.1)									
Moderate/Severe (score 11 to 21)		50	(7.1)	17	(15.9)	4.45	1.58	12.55	**0.005**		4.80	1.43	16.16	**0.011**
**Polysubstance use P6M**	813													
No		352	(49.9)	58	(54.2)									
Yes		354	(50.1)	49	(45.8)	0.61	0.31	1.20	0.152		Not selected			
**Binge drinking measured by Alcohol Use AUDIT-C**	820													
No (Score less than 4)		320	(44.8)	50	(47.6)									
Yes (Score 4 or more)		395	(55.2)	55	(52.4)	0.70	0.36	1.36	0.288					
**IPV experiences**	826													
No		475	(66.2)	88	(81.5)									
Yes		243	(33.8)	20	(18.5)	0.26	0.12	0.58	**0.001**		0.38	0.15	0.95	**0.038**
**Experiences growing up as a child: Sexual abuse**	815													
Never		550	(77.5)	88	(83.8)									
Ever		160	(22.5)	17	(16.2)	0.58	0.22	1.55	0.277					
**Experiences growing up as a child: Touched**	816													
Never		453	(63.7)	75	(71.4)									
Ever		258	(36.3)	30	(28.6)	0.55	0.24	1.30	0.175					
**Experiences growing up as a child: Threatened**	812													
Never		636	(90.0)	96	(91.4)									
Ever		71	(10.0)	9	(8.6)	0.95	0.25	3.55	0.933					
**Experiences growing up as a child: Sexual things**	816													
Never		526	(74.0)	86	(81.9)									
Ever		185	(26.0)	19	(18.1)	0.54	0.21	1.38	0.197					
**Continuous Variables**	**Total N**	**Median**	**(Q1-Q3)**	**Median**	**(Q1-Q3)**	**OR**	**95% CI**		**P-value**					
**P6M Number of male sex partners**	828	8	(4–15)	2	(1–5)	0.92	0.88	0.96	**< 0.001**		Not Selected			
**Treatment Optimism Scale**	828	22	(20–25)	21	(18–25)	0.92	0.86	0.99	**0.028**		0.93	0.85	1.02	0.107
**Age**	828	33	(29–40)	32	(27–36)	0.98	0.94	1.01	**0.182**		0.94	0.89	0.98	**0.009**

Acronyms: HADS = Hospital Anxiety and Depression Scale; P6M = Past Six Months; AUDIT-C = Alcohol Use Disorders Identification Test; IPV = Interpersonal violence; OR = Odds Ratio

*Participants could be included in analysis if they have ever used PrEP before the current visit. Also the mixed effects model considered clustering, so the ORs could not be replicated by the frequencies. Not selected means variables were removed in the model selection process

In addition to syndemic conditions, the time period after the onset of COVID-19 (September 2020-April 2021) was significantly associated with greater odds of PrEP interruptions compared to the time period before COVID-19 (March 2018-March 2020) (aOR = 9.25; 95%CI = 4.06, 21.1). Being in a relationship with a main partner was also positively associated with greater odds of PrEP interruptions (aOR = 3.58; 95%CI = 1.62, 7.92). Furthermore, reporting behaviours that met PrEP eligibility criteria (aOR = 0.25; 95%CI = 0.11, 0.60) and older age (aOR = 0.94; 95%CI = 0.89, 0.98) were associated with lower odds of PrEP interruptions. Greater scores on the treatment optimism-skepticism scale were not significantly associated with PrEP interruptions (aOR = 0.93; 95%CI = 0.85, 1.02). Full results can be found in Table 3.

In our post-hoc analyses assessing self-reported current PrEP use we found overall very similar results to original our analysis assessing PrEP interruptions. GBM who had moderate/severe depression scores had greater odds of not being on PrEP at their interview date based on self-report compared to GBM who had normal/mild scores (aOR = 2.85; 95%CI = 1.24, 6.55). The time period after the onset of COVID-19 (September 2020-April 2021) was significantly associated with greater odds of PrEP not being on PrEP at their interview date compared to the time period before COVID-19 (March 2018-March 2020) (aOR = 1.99; 95%CI = 1.22, 3.26). Being in a relationship with a main partner was also positively associated with greater odds of not being on PrEP at interview date (aOR = 2.99; 95%CI = 1.78, 5.02). Furthermore, reporting behaviours that met PrEP eligibility criteria (aOR = 0.37; 95%CI = 0.21, 0.66) and older age (aOR = 0.97; 95%CI = 0.94, 0.99) were associated with lower odds of not currently being on PrEP at interview date. Greater scores on the treatment optimism-skepticism scale (aOR = 0.89; 95%CI = 0.84, 0.89) and having a greater number of male sex partners (aOR = 0.97; 95%CI = 0.95, 0.99) were also negatively associated with not currently being on PrEP at interview date. Full results can be found in Supplemental Table 1.

## Discussion

We found that the proportion of participants experiencing most syndemic conditions decreased between March 2018 and April 2021, with the exception of moderate/severe depression, where scores increased over time, and moderate/severe anxiety, which were unchanged. Overall, we also found a trend of increased PrEP interruptions over time, with almost a third of ECS GBM on PrEP experiencing an interruption between March-April 2021. However, those who reported sexual behaviours that met eligibility criteria for PrEP enrollment had much lower odds of reporting interrupted PrEP use, suggesting that those most at risk for HIV did not interrupt PrEP treatment. Our findings are aligned with research from other jurisdictions which also noted increased PrEP interruptions since the onset of the COVID-19 pandemic [19, 20, 33]. Moreover, we also noticed a drop in ECS PrEP users since the onset of the COVID-19 pandemic, aligning with national trends from the United States [34]. However, recent data from BC indicate that BC PrEP program engagement declined early in the COVID-19 pandemic, with a partial rebound coinciding with the easing of public health restrictions [35]. In our multivariable model assessing PrEP interruptions, we found moderate/severe depression scores were positively associated and experiences of IPV were negatively associated with experiencing a PrEP interruption. Moreover, we found that the time period post COVID-19 (September 2020-April 2021) was significantly associated with increased odds of PrEP interruptions.

Assessing syndemic trends with categories of before COVID-19 and during COVID-19, we mostly found trends towards fewer reports of syndemic factors, with only moderate/severe depression significantly increasing over time. In all significant trends, there was a noticeable increase/decrease after the start of COVID-19 from September 2020 onwards. Our finding for depression was expected, given the known secondary impacts of COVID-19 on mental health, such as loneliness and isolation [36–38]. Prior to COVID-19, sexual and gender minorities were already disproportionately affected by increased mental health conditions compared to their heterosexual peers [39]. Due to closures of gay bars, queer community spaces, stay at home orders and limiting social gatherings, we suspected that depression and anxiety might increase [40]. Respectively, mixed-methods research among adults who reported a mental health condition in the past year found an increase in reported conditions/diagnoses such as anxiety, obsessive compulsive disorder and increased loneliness [41].

We did not find a significant trend in anxiety scores, which may suggest differences between our GBM sample and the general adult population sample. Additionally, we found decreasing trends for polysubstance use and binge drinking. Although we initially hypothesized that these syndemic conditions might increase, upon further reflection, the observed decreases are understandable. As mentioned previously, public health orders introduced due to the pandemic included the closure of bars, limiting social gatherings and encouraging isolating from others. We suspect that these public health measures may have resulted in reduced alcohol consumption/binge drinking and fewer opportunities for polysubstance use. An online survey of GBM in the US conducted from November 2020 to January 2021 reported significant declines in sexual behaviours such as reductions in willingness to have sex during COVID-19 and a reduced number of condomless anal sex partners [42]. Building off previous research, which found associations between polysubstance use and sexual behaviours (especially group sex events) [43, 44], we hypothesize that reduced sexual behaviours during COVID-19 also resulted in reduced polysubstance use [17].

Overall, we found a trend of increased PrEP interruptions over time. However, our findings should be considered with the fact that PrEP became publicly funded in BC in January 2018 and since then the province has reported general trends of increasing PrEP uptake [27, 45]. Thus, it may be expected that as the number of PrEP users increases, the number of interruptions may also increase. We also found that among the 71 participants who were ever on a PrEP interruption at an interview date during follow-up, over 30% (22 participants) had restarted before the end of follow-up, indicating that, for some, these interruptions were short-lived. We also noticed a reduction in daily PrEP regimens (88.8% vs. 80.4%), increases in on-demand (6.5% vs. 12.1%), and reporting both regimens (4.7% vs. 7.5%) for first visits versus last visits. This finding highlights the potential shift to more flexible PrEP regimens based on changing sexual behaviours during the COVID-19 pandemic.

In our multivariable model, we found depression scores were positively associated with increased odds of experiencing a PrEP interruption, which are aligned with current literature [46]. In a systematic review assessing the PrEP continuum and depression, Miller et al. (2022), found mixed findings on this relationship and point to the non-linear and episodic nature of depressive disorders, which may affect this association [47]. Our study also found that experiences of IPV were associated with lower odds of experiencing a PrEP interruption. In the literature, evidence on experiences of IPV and PrEP use among GBM are limited, as research is mostly among heterosexual women [48]. However, among GBM living with HIV, experiences of IPV have been found to be associated with higher rates of interruptions in care [49]. Disparities in findings between GBM living with HIV and HIV-negative men are worth further exploration. Lastly, given the limited number of significant syndemic conditions in our univariable and multivariable model, we did not find significant interactions between syndemic conditions.

Other key findings from our model were that the time period after the onset of COVID-19 (September 2020-April 2021) was significantly associated with greater odds of PrEP interruptions compared to the time period before COVID-19 (March 2018-March 2020). However, we suspect that differences in PrEP use were associated with changing sexual behaviours and HIV risk. Indeed, qualitative findings from Engage, indicate how GBM adapted sexual practices in response to public health measures and shifting pandemic contexts. These individuals applied their HIV/STI risk mitigation experiences to COVID-19 prevention strategies while engaging in casual sexual behaviours [50]. Importantly, we also found that GBM who met PrEP eligibility criteria at their study visit were less likely to interrupt PrEP compared to GBM who did not meet eligibility. This finding indicates that GBM who were most at risk for HIV were less likely to interrupt treatment, suggesting that GBM who engage in behaviours that place them at greater risk for HIV may understand the benefits of using PrEP and of continuing PrEP as an HIV prevention strategy [51].

This study was subject to a number of strengths and limitations. First, our questionnaire data were self-reported and are subject to social desirability bias. Additionally, PrEP interruptions were considered for once daily dosing only and required six months to determine a PrEP interruption, but participants could have had an on-demand regimen (we found 6.5% self-reported at their first visit and 12.1% at their last visit). However, while a greater than six month interval may seem like a long period, this is the measure that the BC PrEP Program uses to measure PrEP interruptions as many participants use PrEP on an intermittent dosing schedule. As such, the use of shorter gaps to identify PrEP interruptions would likely falsely label many PrEP program participants as interrupting PrEP when they had not. Overall, a major strength of this study is the direct linkages to the BC PrEP program, which distributes PrEP medication in the province. Thus, we were able to identify PrEP interruptions directly, instead of relying on self-reported data. Second, along with many sexual health clinics during this time, our study offices were closed from March 2020-September 2020, resulting in missing survey data from that period. Coincidentally, most items from our questionnaire, including substance use, IPV, and AUDIT-C scale ask about experiences in the past six months. Therefore, if participants were missing data from that time, we were able to infer some of their experiences using the six month lookback time. Third, our study recruited urban GBM living in the Metro Vancouver area using RDS and may not be representative of all GBM. RDS recruitment is based on social networks and GBM who are not connected with the lesbian, gay, bisexual, transgender, 2-Spirit and queer (2SLGBT2Q+) communities or are isolated may be underrepresented. However, a strength of RDS is the ability to recruit a more probabilistic community-based sample of GBM and the collection of longitudinal data. Overall, this research provides a window on the health and wellbeing of GBM during the COVID-19 pandemic and highlights areas to improve services to better support this population.

## Conclusion

This research highlights how the secondary effects of the COVID-19 pandemic affected the health and wellbeing of HIV-negative/unknown GBM in Vancouver, Canada. While increasing PrEP interruptions are concerning, we found that GBM most at risk were also less likely to interrupt treatment. Among GBM who did interrupt treatment, future research should examine how long these interruptions lasted and factors associated with restarting treatment. Moreover, findings on associations between depression and PrEP interruptions suggest future interventions should consider additional mental health services and targeted follow-up for PrEP continuation to mitigate the impacts of the COVID-19 pandemic on GBM.

### Electronic supplementary material

Below is the link to the electronic supplementary material.

Supplementary Material 1

## Data Availability

Data and materials are available from the corresponding author upon reasonable request.

## Declarations

or comparable ethical standards. Informed consent was obtained from all individual participants included in the study.
