# Supplementary material for: Examining the secondary impacts of the COVID-19 pandemic on syndemic production and PrEP use among gay, bisexual and other men who have sex with men (GBM) in Vancouver, Canada
Source: BMC Public Health. 2023 Oct 30;23:2124. doi: 10.1186/s12889-023-17049-w (PMC10614320; doi:10.1186/s12889-023-17049-w)
Supplement: Supplementary file 1 — Supplementary Material 1 [file 12889_2023_17049_MOESM1_ESM.docx]

**Supplemental File**

**Table 1: Univariable and Multivariable Generalized linear mixed Models Assessing Self-Reported Current PrEP Use* (N=314 participants reporting on 926 visits)**

| **Self-Reported not currently on PrEP** | | | | | | | | | | | | | | | | | |
| --- | --- | --- | --- | --- | --- | --- | --- | --- | --- | --- | --- | --- | --- | --- | --- | --- | --- |
|  |  | **No (N=661)** | | **Yes (N=265)** | | **Yes vs. No** | | | | **Yes vs. No** | | | | | | | |
| **Variable** | **Total N** | **N** | **(%)** | **N** | **(%)** | **OR** | **95% CI** | | **p-value** | | **aOR** | | **95% CI** | | | **p-value** | |
| **Annual income** | 926 |  |  |  |  |  |  |  |  | |  | |  |  | |  | |
| Less than 30000 |  | 174 | (26.3) | 85 | (32.1) |  |  |  |  | |  | |  |  | |  | |
| 30000 to 59999 |  | 247 | (37.4) | 91 | (34.3) | 0.84 | 0.47 | 1.48 | 0.541 | |  | |  |  | |  | |
| 60000 or higher |  | 240 | (36.3) | 89 | (33.6) | 0.81 | 0.43 | 1.50 | 0.497 | |  | |  |  | |  | |
| **Ethnicity** | 926 |  |  |  |  |  |  |  |  | |  | |  |  | |  | |
| Canadian |  | 286 | (43.3) | 130 | (49.1) |  |  |  |  | |  | |  |  | |  | |
| Aboriginal |  | 5 | (0.8) | 4 | (1.5) | 3.19 | 0.25 | 40.26 | 0.37 | | Not Selected | |  |  | |  | |
| European |  | 170 | (25.7) | 53 | (20.0) | 0.63 | 0.32 | 1.24 | 0.177 | |  | |  |  | |  | |
| Asian |  | 131 | (19.8) | 42 | (15.8) | 0.58 | 0.27 | 1.24 | 0.161 | |  | |  |  | |  | |
| African/Caribbean/Black |  | 10 | (1.5) | 1 | (0.4) | 0.13 | 0.01 | 2.39 | 0.169 | |  | |  |  | |  | |
| Mixed Race |  | 14 | (2.1) | 8 | (3.0) | 1.59 | 0.24 | 10.53 | 0.628 | |  | |  |  | |  | |
| Another ethnicity |  | 45 | (6.8) | 27 | (10.2) | 1.72 | 0.63 | 4.67 | 0.288 | |  | |  |  | |  | |
| **Sexual identity** | 926 |  |  |  |  |  |  |  |  | |  | |  |  | |  | |
| Gay |  | 563 | (85.2) | 222 | (83.8) |  |  |  |  | |  | |  |  | |  | |
| Bisexual |  | 23 | (3.5) | 5 | (1.9) | 0.52 | 0.12 | 2.25 | 0.383 | |  | |  |  | |  | |
| Another sexual identity |  | 75 | (11.3) | 38 | (14.3) | 1.12 | 0.55 | 2.28 | 0.755 | |  | |  |  | |  | |
| **Gender identity** | 926 |  |  |  |  |  |  |  |  | |  | |  |  | |  | |
| Cisgender |  | 623 | (94.3) | 246 | (92.8) |  |  |  |  | |  | |  |  | |  | |
| Another gender identity |  | 38 | (5.7) | 19 | (7.2) | 1.26 | 0.48 | 3.29 | 0.644 | |  | |  |  | |  | |
| **Highest level of education** | 926 |  |  |  |  |  |  |  |  | |  | |  |  | |  | |
| High school or less |  | 87 | (13.2) | 44 | (16.6) |  |  |  |  | |  | |  |  | |  | |
| Greater than high school |  | 574 | (86.8) | 221 | (83.4) | 0.61 | 0.31 | 1.17 | 0.136 | | 0.53 | | 0.26 | 1.06 | | 0.074 | |
| **Current employment** | 926 |  |  |  |  |  |  |  |  | |  | |  |  | |  | |
| No |  | 107 | (16.2) | 46 | (17.4) |  |  |  |  | |  | |  |  | |  | |
| Yes |  | 554 | (83.8) | 219 | (82.6) | 0.90 | 0.50 | 1.63 |  | |  | |  |  | |  | |
| **Current relationship with a main partner** | 926 |  |  |  |  |  |  |  |  | |  | |  |  | |  | |
| No |  | 367 | (55.5) | 120 | (45.3) |  |  |  |  | |  | |  |  | |  | |
| Yes |  | 294 | (44.5) | 145 | (54.7) | 2.35 | 1.45 | 3.79 | **0.001** | | 2.99 | | 1.78 | 5.02 | | **<0.001** | |
| **Time period** | 926 |  |  |  |  |  |  |  |  | |  | |  |  | |  | |
| Before COVID (MAR 2018 to MAR 16 2020) |  | 518 | (78.4) | 173 | (65.3) |  |  |  |  | |  | |  |  | |  | |
| After COVID (SEP 2020 to APR 2021) |  | 143 | (21.6) | 92 | (34.7) | 2.91 | 1.86 | 4.56 | **<0.001** | | 1.99 | | 1.22 | 3.26 | | **0.006** | |
| **Overall PrEP eligibility** | 921 |  |  |  |  |  |  |  |  | |  | |  |  | |  | |
| No |  | 102 | (15.5) | 81 | (30.7) |  |  |  |  | |  | |  |  | |  | |
| Yes |  | 555 | (84.5) | 183 | (69.3) | 0.29 | 0.17 | 0.49 | **<0.001** | | 0.37 | | 0.21 | 0.66 | | **0.001** | |
| **Syndemic Conditions** |  |  |  |  |  |  |  |  |  | |  | |  |  | |  | |
| **HADS Anxiety** | 910 |  |  |  |  |  |  |  |  | |  | |  |  | |  | |
| Normal/Mild (score 10 or less) |  | 498 | (76.6) | 185 | (71.2) |  |  |  |  | |  | |  |  | |  | |
| Moderate/Severe (score 11 to 21) |  | 152 | (23.4) | 75 | (28.8) | 1.59 | 0.95 | 2.65 | 0.076 | | Not Selected | |  |  | |  | |
| **HADS Depression** | 907 |  |  |  |  |  |  |  |  | |  | |  |  | |  | |
| Normal/Mild (score 10 or less) |  | 602 | (93.6) | 236 | (89.4) |  |  |  |  | |  | |  |  | |  | |
| Moderate/Severe (score 11 to 21) |  | 41 | (6.4) | 28 | (10.6) | 2.99 | 1.32 | 6.78 | **0.009** | | 2.85 | | 1.24 | 6.55 | | **0.014** | |
| **Polysubstance use P6M** | 910 |  |  |  |  |  |  |  |  | |  | |  |  | |  | |
| No |  | 306 | (47.2) | 142 | (54.2) |  |  |  |  | |  | |  |  | |  | |
| Yes |  | 342 | (52.8) | 120 | (45.8) | 0.56 | 0.34 | 0.91 | **0.02** | | Not selected | |  |  | |  | |
| **Binge drinking measured by Alcohol Use AUDIT-C** | 918 |  |  |  |  |  |  |  |  | |  | |  |  | |  | |
| No (Score less than 4) |  | 310 | (47.2) | 119 | (45.6) |  |  |  |  | |  | |  |  | |  | |
| Yes (Score 4 or more) |  | 347 | (52.8) | 142 | (54.4) | 1.02 | 0.63 | 1.65 | 0.94 | |  | |  |  | |  | |
| **IPV experiences** | 924 |  |  |  |  |  |  |  |  | |  | |  |  | |  | |
| No |  | 433 | (65.7) | 185 | (69.8) |  |  |  |  | |  | |  |  | |  | |
| Yes |  | 226 | (34.3) | 80 | (30.2) | 0.67 | 0.41 | 1.07 | 0.092 | | Not selected | |  |  | |  | |
| **Experiences growing up as a child: Sexual abuse** | 913 |  |  |  |  |  |  |  |  | |  | |  |  | |  | |
| Never |  | 511 | (78.3) | 209 | (80.4) |  |  |  |  | |  | |  |  | |  | |
| Ever |  | 142 | (21.7) | 51 | (19.6) | 0.86 | 0.44 | 1.67 | 0.654 | |  | |  |  | |  | |
| **Experiences growing up as a child: Touched** | 914 |  |  |  |  |  |  |  |  | |  | |  |  | |  | |
| Never |  | 421 | (64.5) | 171 | (65.5) |  |  |  |  | |  | |  |  | |  | |
| Ever |  | 232 | (35.5) | 90 | (34.5) | 0.55 | 0.24 | 1.30 | 0.175 | |  | |  |  | |  | |
| **Experiences growing up as a child: Threatened** | 910 |  |  |  |  |  |  |  |  | |  | |  |  | |  | |
| Never |  | 584 | (90.0) | 236 | (90.4) |  |  |  |  | |  | |  |  | |  | |
| Ever |  | 65 | (10.0) | 25 | (9.6) | 1.06 | 0.44 | 2.57 | 0.897 | |  | |  |  | |  | |
| **Experiences growing up as a child: Sexual things** | 914 |  |  |  |  |  |  |  |  | |  | |  |  | |  | |
| Never |  | 482 | (73.8) | 196 | (75.1) |  |  |  |  | |  | |  |  | |  | |
| Ever |  | 171 | (26.2) | 65 | (24.9) | 0.92 | 0.50 | 1.70 | 0.793 | |  | |  |  | |  | |
| **Continuous Variables** | **Total N** | **Median** | **(Q1-Q3)** | **Median** | **(Q1-Q3)** | **OR** | **95% CI** | | **P-value** | |  | |  |  | |  | |
| **P6M Number of male sex partners** | 926 | 9 | (4-20) | 3 | (1-8) | 0.94 | 0.92 | 0.96 | **<0.001** | | 0.97 | 0.95 | | | 0.99 | | **0.003** |
| **Treatment Optimism Scale** | 926 | 23 | (20-26) | 21 | (18-24) | 0.88 | 0.84 | 0.93 | **<0.001** | | 0.89 | | 0.84 | 0.94 | | **<0.001** | |
| **Age** | 926 | 34 | (29-41) | 31 | (28-38) | 0.98 | 0.96 | 1.00 | 0.101 | | 0.97 | | 0.94 | 0.99 | | **0.017** | |

*Acronyms: HADS=Hospital Anxiety and Depression Scale; P6M=Past Six Months; AUDIT-C=Alcohol Use Disorders Identification Test; IPV=Interpersonal violence; OR=Odds Ratio*

**Participants could be included in analysis if they have ever used PrEP before the current visit. Also the mixed effects model considered clustering, so the ORs could not be replicated by the frequencies. Not selected means variables were removed in the model selection process.*

*Variable is Self-reported not currently on PrEP to keep OR in the same direction as previous model*
